# Evaluation of SARS-CoV-2 Spike S1 Protein Response on PI3K-Mediated IL-8 Release

**DOI:** 10.3390/medsci9020030

**Published:** 2021-05-18

**Authors:** Christina Borchers, Anita Thyagarajan, Christine M. Rapp, Jeffrey B. Travers, Ravi P. Sahu

**Affiliations:** 1 Department of Pharmacology and Toxicology, Boonshoft School of Medicine, Wright State University, Dayton, OH 45345, USA; christina.borchers@wright.edu (C.B.); anita.thyagarajan@wright.edu (A.T.); christine.rapp@wright.edu (C.M.R.); jeffrey.travers@wright.edu (J.B.T.); 2 Department of Dermatology, Wright State Physicians, Wright State University, Dayton, OH 45345, USA

**Keywords:** COVID-19, PI3K signaling, interleukin-8

## Abstract

A novel coronavirus related to a condition known as a severe acute respiratory syndrome (SARS) was termed as SARS Coronavirus-19 (SARS-CoV-2 or COVID-19), which has caused an unprecedented global pandemic. Extensive efforts have been dedicated worldwide towards determining the mechanisms of COVID-19 associated pathogenesis with the goals of devising potential therapeutic approaches to mitigate or overcome comorbidities and mortalities. While the mode of SARS-CoV-2 infection, its structural configuration, and mechanisms of action, including the critical roles of the Spike protein have been substantially explored, elucidation of signaling pathways regulating its cellular responses is yet to be fully determined. Notably, phosphoinositide 3-kinases (PI3K) and its downstream pathway have been exploited among potential therapeutic targets for SARS-CoV-2, and its activation modulates the release of cytokines such as IL-8. To that end, the current studies were sought to determine the response of the SARS-CoV-2 Spike S1 protein on PI3K-mediated IL-8 release using relevant and widely used cellular models. Overall, these studies indicate that PI3K signaling does not directly mediate Spike S1 protein-induced IL-8 release in these cellular models.

## 1. Introduction

The SARS-CoV-2 outbreak (also referred to as COVID-19) started in December 2019 and was later declared by The World Health Organization (WHO) as a global pandemic, which has affected over 66 million people worldwide until early December 2020, resulting in a death toll surpassing 1.5 million cases. Of significance, older people (>65 years) and patients with predisposing medical conditions, including diabetes, cardiovascular, or lung diseases, have been found to be at a higher risk of more likely developing serious symptoms/complications, leading to an increased mortality rate [1,2,3,4]. Meanwhile, the overall risks and death rates in non-elderly individuals (<65 years old) were reported to be less compared to the non-elderly people with comorbidities [5]. Moreover, healthcare workers, young adults, and the pediatric population have also been found susceptible to SARS-CoV-2 infection [6,7,8]. However, the majority of these individuals either remained asymptomatic or developed mild-to-moderate symptoms, including fever, dry cough, and respiratory illness [6,7,8].

Multiple underlying pathogeneses, including altered immune responses, immune suppression, a state of chronic systemic inflammation associated with cytokine storm, lung injury, and multiorgan system failure, are associated with poor prognosis in SARS-CoV-2 contracted individuals, which subsequently increased their morbidity and mortality [9,10,11]. To that end, extensive efforts have been dedicated worldwide towards determining the mechanisms of COVID-19 associated pathogenesis with the goals of devising potential therapeutic approaches to mitigate or overcome comorbidities and mortalities. Several immunological and pharmacological interventions have been explored for treating COVID-19 patients, which were associated with encouraging outcomes and reduced mortality [12,13,14,15]. Besides, there are no specific drugs for COVID-19 treatment or to control the cytokine storm leading to rapid disease progression. However, many clinical trials are currently testing vaccines in diverse settings of the human population, and their results are underway. Moreover, in addition to the restrictions implemented by the US Centers for Disease Control and Prevention (CDC), several preventative supplementation approaches, including nutraceuticals and vitamin D, have been suggested to help strengthen the immune system to avoid or overcome infections [16,17].

Notably, much has been explored in terms of the SARS-CoV-2 infection and the specific role of the angiotensin-converting enzyme 2 (ACE2) receptor, a part of the dual renin-angiotensin-system (RAS) in facilitating its entry to a wide range of human cells [18]. This exploration has also rationalized the evaluation of ACE inhibitors in COVID-19 patients [19,20]. Besides, the interaction between the ACE2 receptor and the SARS-CoV-2 Spike protein has been shown to play a critical role in host cell recognition, which determines the overall disease severity [21,22]. However, the elucidation of critical signaling pathways involved in regulating SARS-CoV-2 Spike protein-mediated cellular responses is yet to be fully determined. Importantly, among various cellular pathways, phosphoinositide 3-kinase (PI3K)/protein kinase B (PKB, also known as AKT) and its downstream molecule, mammalian target of rapamycin (mTOR), are considered as the potential therapeutic targets against SARS-CoV-2 [23,24], and their activation modulates cytokine release such as interleukin 8 (IL-8) [25,26].

To that end, the current studies were sought to determine the response of the SARS-CoV-2 Spike S1 protein (referred to as Spike S1) on PI3K-mediated IL-8 release via phorbol 12-myristate 13-acetate (PMA) using relevant and widely used cellular models. The rationale of using PMA is supported by several studies demonstrating that PI3K activation is required for PMA-induced effects [27,28,29]. This is due to the crosstalk between PI3K/AKT and protein kinase C (PKC, for which the PMA acts as an agonist) pathways, as inhibitors of PI3K have been shown to block PMA-induced effects [27,28,29]. Our studies demonstrate that only PMA but not Spike S1 were able to induce IL-8 release. Moreover, the pretreatment of PMA but not Spike S1 elicited an increased IL-8 secretion. Overall, these studies indicate that PI3K signaling does not directly mediate Spike S1-induced IL-8 release in these cellular models.

## 2. Materials and Methods

### 2.1. Reagents and Cell Lines

The PMA was purchased from Sigma-Aldrich (St. Louis, MO, USA). The SARS-CoV-2 Spike S1 subunit protein (synonyms: Spike protein, S Protein, S1 Subunit, host cell receptor-binding domain (RBD)) was from RayBiotech (Peachtree Corners, GA, USA). The human IL-8 ELISA kit was procured from R&D Systems (Minneapolis, MN, USA). The culture media DMEM was purchased from Corning Mediatech, Inc. (Manassas, VA, USA), and F-12K was from GE Healthcare Biosciences (Marlborough, MA, USA). The fetal bovine serum (FBS) was from Corning (Corning, NY, USA), antibiotic–antimycotic was from Gibco (Gaithersburg, MD, USA), and penicillin–streptomycin was purchased from Hyclone (Logan, UT, USA). Human nasopharyngeal carcinoma KBP cells were grown in DMEM high glucose media, and non-small cell lung cancer A549 cells were grown in F-12K media supplemented with 10% FBS and antibiotic/antimycotic, as previously described [30,31].

### 2.2. IL-8 Release

Cell lines were grown to approximately 80–90% confluency in 6 well plates and then treated with various doses of PMA, Spike S1 subunit protein, or a combination of PMA and Spike S1, mentioned in the figure legends. Following incubations at the given time points, the supernatants were collected and tested for IL-8 secretion using a human IL-8 ELISA kit, similar to as previously reported [30,32].

### 2.3. Statistical Analysis

Statistical analysis was assessed by GraphPad Prism software version 7.0 (GraphPad Software, San Diego, CA, USA). The experiments were repeated, independently, at least three times. Data were analyzed by Student’s *t*-test (to compare between two groups) or one-way ANOVA (for more than two groups) with post hoc Tukey or Bonferroni multiple comparison tests. The value of *p* < 0.05 was considered to indicate a statistically significant difference between the tested groups.

## 3. Results and Discussion

### 3.1. Evaluation of Spike S1 Response on IL-8 Release

**Figure 1:**
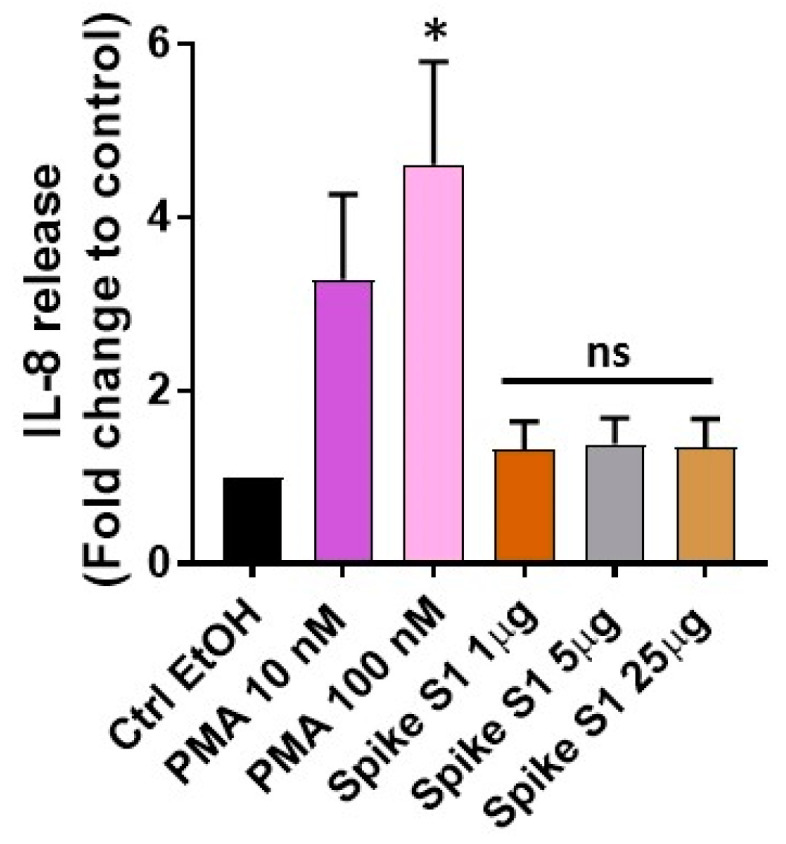
Dose–response evaluation of PMA and Spike S1 on IL-8 release. KBP cells were treated with 0.1% ethanol (EtOH) as vehicle control (Ctrl-EtOH) or with various concentrations of PMA (10 and 100 nM) and Spike S1 (1, 5, and 25 µg/mL). After 6 h of incubation, the supernatants were collected and evaluated for IL-8 secretion by ELISA assay. Data are mean ± SE from three independent experiments done in triplicates, normalized per 1 × 10^6^ cells, and represented as IL-8 release (fold change to control). A statistically significant difference (* = *p* < 0.05) was observed between Ctrl-EtOH and PMA (100 nM), and ns denotes non-significant differences between the Ctrl-EtOH- and Spike S1-treated groups.

Multiple cell types such as the epithelial lining of the nasal, tracheobronchial, bronchial, and respiratory cells have been shown to be primarily infected by SARS-CoV-2 [33,34]. To that end, our first studies tested the dose–response effect of Spike S1 on IL-8 release from human nasopharyngeal carcinoma and the KBP cell line using PMA as a positive control. For this, we took advantage of our published report indicating that PMA (100 nM dose) induces IL-8 secretion from KBP cells [32]. As shown in Figure 1, PMA induces a dose-dependent release of IL-8 as compared to the vehicle control (i.e., Ctrl-EtOH)-treated group. However, we did not notice IL-8 release by Spike S1 at all the concentrations evaluated as compared to the Ctrl-EtOH group (Figure 1).

Given that SARS-CoV-2 infection and its severity in various experimental models and humans have been found to be dependent on the dose/load of this virus [35,36,37], we considered the fact that the Spike S1 doses used in our study might not be sufficient to induce an IL-8 response. However, a recent report demonstrated that the SARS-CoV-2 spike protein enhanced ACE2 activity (i.e., ACE2 proteolytic activity was measured via the degradation of fluorogenic caspase-1 substrate and ACE2 cleavage of bradykinin analog) in a dose-dependent manner with significant changes noted at 7, 14, and 75 µg/mL [38]. In addition, only the SARS-CoV-2 RBD but not the SARS-CoV RBD was found to enhance ACE2 activity, indicating the specific response of the Spike S1 protein (RBD) in augmenting SARS-CoV-2-induced ACE2 activity.

Along similar lines, studies by Bortolotti and colleagues have shown that the SARS-CoV-2 Spike 1 protein (at 1 µg concentration) transfected lung epithelial cells and modifies the degranulation and cytotoxicity of co-cultured natural killer (NK) cells [39]. NK cell degranulation was measured via CD107a staining and cytotoxicity using a 7AAD/CFSE Cell-mediated cytotoxicity assay kit. These findings indicate that the Spike S1 protein concentrations used in our study were within the range (Figure 1) that was able to induce the functional responses in ACE2 and NK cell activities. Thus, the observed differences noted in the cellular responses of the SARS-CoV-2 Spike 1 protein could be due to the different model systems (e.g., FreeStyle293 F cells [38], K562 lymphoblastoid, and BEAS-2B bronchial lung epithelial cell lines [39] versus KBP and A549 (as detailed below) cell lines).

### 3.2. Effects of PMA, Spike S1, and Their Combination on IL-8 Secretion

**Figure 2:**
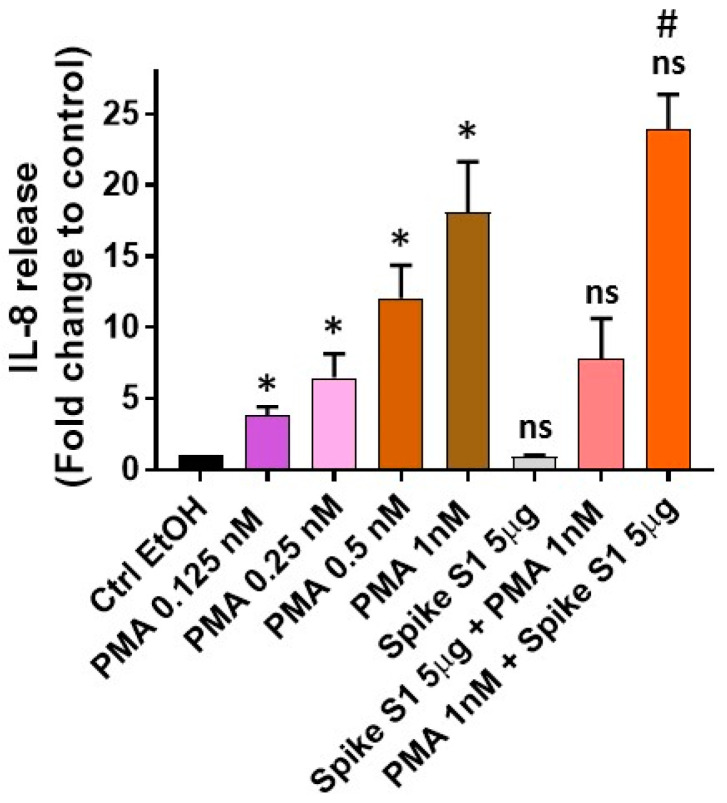
Evaluations of the dose–response effect of PMA and its combination with Spike S1 on IL-8 release. A549 cells were treated with 0.1% EtOH as vehicle control (Ctrl-EtOH), with various doses of PMA (0.125, 0.25, 0.5, and 1 nM), Spike S1 (5 µg/mL), and a combination of Spike S1 and PMA or PMA and Spike S1. After 24 h of incubation for PMA and Spike S1 alone treatments or 30 h of incubation for combination treatments, the supernatants were collected and evaluated for IL-8 secretion by ELISA assay. Data are mean ± SE from three independent experiments done in triplicates, normalized per 1 × 10^6^ cells, and represented as IL-8 release (fold change to control). Statistically significant differences were observed between Ctrl-EtOH and PMA (* = *p* < 0.05), and Spike S1 vs. PMA + Spike S1 (# = *p* < 0.01). ns denotes non-significant differences were observed between Ctrl-EtOH and Spike S1, PMA vs. Spike S1 + PMA or PMA + Spike S1, and Spike S1 vs. Spike S1 + PMA.

Given that A549, a human non-small cell lung cancer (NSCLC) cell line of alveolar origin has also been shown to be infected by SARS-CoV-2 [34], we attempted to evaluate its response on IL-8 secretion. For this, we tested a similar dose–response effect of PMA (as in Figure 1) along with Spike S1 at a dose of 5 µg/mL, as a similar trend of IL-8 release was noted at all the concentrations (Figure 1). We observed a much higher dose–response of PMA on IL-8 release (data not shown). Thus, our next studies evaluated the PMA dose–response effect at much lower concentrations (ranging from 0.125 to 1 nM). As shown in Figure 2, we observed that PMA induces IL-8 release in a dose-dependent manner, yet no response of Spike S1 was noted as compared to the Ctrl-EtOH group.

To determine if pre-stimulation with Spike S1 or PMA (1 nM) could elicit higher IL-8 secretion, we tested the effects of Spike S1 and PMA, as well as PMA and Spike S1 combinations. Our studies demonstrate that there were no significant differences in Spike S1 + PMA or PMA + Spike S1 combinations when compared with the PMA alone group (1 nM) (Figure 2). Moreover, no significant difference was noted between the Spike S1 + PMA combination and the Spike S1 alone group. However, significantly increased IL-8 release was noticed by the PMA + Spike S1 combination when compared with the Spike S1 alone group (Figure 2).

These findings indicate that pretreatment of PMA but not Spike S1 elicits an increased IL-8 secretion, which is not surprising given that PMA alone (1nM) treatment exhibited a significantly higher response of IL-8 release (Figure 2). Overall, these studies indicate that PI3K signaling does not directly mediate Spike S1-induced IL-8 release in these cellular models despite the fact that nasopharyngeal carcinomas and the A549 cell line have been found to express high levels of ACE2 expression [40,41,42]. Besides, there are several limitations to these studies. First, only two cell lines (KBP and A549) were tested for Spike S1 protein response on IL-8 release. Second, future studies are required to further determine the role of other SARS-CoV-2 Spike proteins in the same context. Finally, other cellular models of tracheobronchial or bronchial epithelial origins such as Calu-3 and primary alveolar type II (ATII) cell lines should be explored to evaluate SARS-CoV-2 Spike protein responses, which have been shown to be permissive to SARS-CoV-2 infection [34,43].

Several studies have shown that the SARS-CoV-2 spike (S) glycoprotein binds with ACE2 with higher affinity and that the S1 subunit containing a receptor-binding domain (RBD) of one protomer in the spike protein trimer tightly interacts with ACE2 extracellular enzymatic domain [38,44,45,46,47]. To that end, high-affinity peptide sequences and neutralizing antibodies targeting SARS-CoV-2 spike-RBD have been developed with the overall goal of evaluating their efficacy as novel diagnostic or therapeutic modalities [48,49,50]. Thus, it is important to explore the critical signaling pathways involved in regulating the spike protein to gain further mechanistic insights to develop novel therapies for COVID-19 treatment.

## Data Availability

Not applicable.
